# Psychotropic medication prescribing for patients with insomnia comorbid with depressive or anxiety disorders in primary healthcare facilities in Beijing

**DOI:** 10.1192/bjo.2025.10967

**Published:** 2026-02-05

**Authors:** Mengyuan Fu, Can Li, Xinyi Zhou, Zhiwen Gong, Yuezhen Zhu, Yingtian Ding, Kexin Ling, Fang Wang, Luwen Shi, Xiaodong Guan

**Affiliations:** International Research Center for Medicinal Administration, Peking University, Beijing, China; Department of Internal Medicine, Yale School of Medicine, New Haven, Connecticut, USA; Department of Pharmacy Administration and Clinical Pharmacy, School of Pharmaceutical Sciences, Peking University, Beijing, China; Department of Pharmacy, Beijing Chao-Yang Hospital, Capital Medical University, Chaoyang District, Beijing, China; Department of Pharmacy Administration, Dongcheng Health Service Management Center, Beijing, China

**Keywords:** Insomnia, comorbid depressive disorders, comorbid anxiety disorders, psychotropic medications, guideline-recommended pharmacotherapy

## Abstract

**Background:**

Depressive and anxiety disorders often co-occur with insomnia, creating complex treatment challenges. Although clinical guidelines recommend psychotherapy as first-line treatment for these comorbid conditions, limited access to psychological services in primary healthcare facilities in China often leads to heavy reliance on pharmacological therapy.

**Aims:**

To the appropriateness of psychotropic medications for patients with insomnia comorbid with depressive or anxiety disorders at primary healthcare facilities in China.

**Method:**

This cross-sectional study included patients with documented diagnoses of insomnia comorbid depressive or anxiety disorders in 2022 at all 67 primary healthcare facilities in Dongcheng District, Beijing, China. The primary outcome was the prescribing rate of guideline-recommended psychotropic medications.

**Results:**

Among 842 patients with insomnia and depressive disorders and 1014 patients with insomnia and anxiety disorders, over 90% received psychotropic medications. Benzodiazepines were the most frequently prescribed classes (55.9 and 69.6%), followed by non-benzodiazepine hypnotics (42.5 and 42.4%), whereas medications recommended by the guideline, including antidepressants with sedative effects, selective serotonin reuptake inhibitors and selective norepinephrine reuptake inhibitors, were used infrequently. Only 29.9% of patients with insomnia and depressive disorders and 11.5% of those with insomnia and anxiety disorders received guideline-recommended pharmacotherapy, with lower concordance among older adults.

**Conclusions:**

Guideline-recommended pharmacotherapy for insomnia comorbid with depressive or anxiety disorders was rarely implemented at primary care in China. This highlights the need to facilitate evidence-based practices and improve management of comorbid mental health conditions, particularly for older adults.

Depressive and anxiety disorders are both associated with insomnia.^1^ Studies indicate that over 70% of individuals with major depressive disorder and 50% of those with anxiety disorder experience insomnia.^2^ Conversely, insufficient sleep can also precipitate or even exacerbate various mental disorders.^3^ Individuals enduring persistent insomnia report a 65% recurrence rate of depression, compared with 13% for those without insomnia.^4^ Despite these associations, concurrent management of insomnia alongside depression and anxiety is often overlooked in clinical practice.^5,6^ Current expert consensus and clinical guidelines recommend a dual-focus approach when treating these comorbid conditions, and emphasise the importance of addressing both the mental disorders and insomnia, as their coexistence presents distinct clinical manifestations, treatment strategies and prognoses compared with insomnia alone.^5,7^

## Guideline recommendations

Cognitive–behavioural therapy (CBT) is recognised as the first-line treatment for both insomnia and common mental disorders. For chronic insomnia, current guidance recommends CBT for insomnia as the first-line treatment and reserves pharmacologic agents, particularly non-benzodiazepine hypnotics and benzodiazepines, for short-term or refractory use.^8^ For depressive disorder, stepped-care guidance recommends psychological therapies (e.g. CBT) as the first-line treatment for mild to moderate episodes, whereas moderate to severe episodes generally warrant antidepressant pharmacotherapy, with or without psychotherapy.^9^ For anxiety disorders (e.g. generalised anxiety disorder), disorder-specific CBT is the first-line treatment.^10^ However, a lack of resources and trained professionals have resulted in limited availability of CBT, especially at primary healthcare facilities in China,^11,12^ and so pharmacological interventions are commonly and often inappropriately used.^13,14^ Approximately 15% of adult out-patient visits at primary healthcare facilities involved a prescription for insomnia.^14^ Chinese clinical guideline recommends selective serotonin reuptake inhibitors (SSRIs), selective norepinephrine reuptake inhibitors (SNRIs) and antidepressants with sedative effects (e.g. mirtazapine, trazodone) as the first-line pharmacological options for patients with insomnia comorbid with depressive or anxiety disorders, while reserving non-benzodiazepine hypnotics (also known as Z-drugs) as supplementary medications and benzodiazepines as a last resort.^7^

## Primary care evidence gap

Primary care is pivotal in the management of common mental disorders.^15,16^ Although prescribing practices for psychotropic medications, such as benzodiazepines, non-benzodiazepine, anxiolytics and antidepressants, have been extensively studied in high-income countries,^17–19^ the appropriateness of their use among patients with insomnia comorbid with common mental disorders at primary healthcare facilities remains underexplored in China. Therefore, we designed this study to evaluate the prescribing rate and appropriateness of psychotropic medications at primary healthcare facilities for patients experiencing insomnia with comorbid depressive or anxiety disorders.

## Method

### Study design

We conducted a cross-sectional study at all 67 primary healthcare facilities in Dongcheng district, Beijing, China, in 2022. We also assessed the appropriateness of pharmacotherapy based on China’s guideline for the diagnosis and treatment of insomnia comorbid with depression or anxiety in adults.^7^ Ethical approval for this study was obtained from the Peking University Institution Review Board (approval number IRB00001052-17016). A waiver of informed consent was granted because the data were deidentified. The study followed the Strengthening the Reporting of Observational Studies in Epidemiology (STROBE) reporting guideline for observational studies.^20^

### Sampling and data collection

Patients identified with insomnia (ICD-10-CM codes F51.0X and G47.0X), combined with depressive disorders (ICD-10-CM codes F32.X, F33.X, F34.1) or anxiety disorders (ICD-10-CM codes F41.X, F40) documented in their diagnoses in 2022 were eligible for inclusion in our study.^21^ To improve diagnostic accuracy, we also cross-checked the corresponding Chinese text diagnoses recorded in the electronic health records, to confirm the coding and reduce potential misclassification. Given that primary clinicians may perceived insomnia as a secondary symptom of depressive and anxiety disorders, and the treatment duration for these mental disorders often extends over prolonged periods with common recurrences,^22,23^ patients diagnosed with these conditions in separate visits within the same year were also considered comorbid. We extracted data on out-patient visit dates, patient demographics, diagnoses and prescribed medications from the electronic health records. All data were digitally transferred and verified.

### Definition

This study categorised anxiolytics, hypnotics and sedatives, antidepressants and antipsychotics as psychotropic medications, using the Anatomical Therapeutic Chemical classification (Supplementary Table 1 available at https://doi.org/10.1192/bjo.2025.10967).^24^ We defined guideline-recommended pharmacotherapy based on Chinese guidelines.^7,8^ A concise mapping of recommendations to medication classes appears in Supplementary Table 2. Specifically, SSRIs, SNRIs and antidepressants with sedative effects (i.e. mirtazapine, trazodone) are recommended as first-line pharmacotherapies for patients with insomnia comorbid with depressive or anxiety disorders. For patients with insomnia and depression, non-benzodiazepines, should be preferred over benzodiazepines for short-term use when necessary. For those with both insomnia and anxiety, benzodiazepines are recommended for short-term use when insomnia symptoms are more prominent. The fixed-dose combination of flupentixol and melitracen is also recommended by Chinese guidelines.^7^ We focused on all visits that contained at least one psychotropic medication and categorised them into three groups based on guideline recommendations: recommended, conditional recommended, and not recommended or not mentioned.

### Outcome measures

The primary outcome was the prescribing rate of appropriate psychotropic medications for treating insomnia combined with depressive or anxiety disorders across different age groups. This measure was calculated as the proportion of patient visits that were prescribed with guideline-recommended pharmacotherapy out of total patient visits that were prescribed with at least one psychotropic medication. The second outcome was the prescribing rate of psychotropic medications across different drug classes.

### Statistical analysis

We used descriptive statistics to summarise patient characteristics and outcome measures. Continuous variables were reported with means and s.d., whereas categorical variables were expressed as counts and proportions, stratified by patient age group. Statistical analyses were performed with Stata BE (version 18.0 for Windows; StataCorp LLC, College Station, Texas, USA; https://www.stata.com).

## Results

### Study sample and characteristics

We identified 55 320 adult patients with a diagnosis of insomnia in primary healthcare facilities in Beijing in 2022. Of these, 842 (1.5%) patients had comorbid depressive disorders and 1014 (1.8%) had comorbid anxiety disorders (Supplementary Fig. 1). The mean ages of these two groups were 68.8 (s.d. 12.3) and 66.5 (s.d. 12.3), respectively. The study sample predominantly consisted of women (62.1% for insomnia comorbid depression and 63.2% for insomnia comorbid anxiety) and individuals covered by medical insurance (95.2% for insomnia comorbid depression and 95.3% for insomnia comorbid anxiety). The most common comorbidities were hypertension (66.6% for insomnia comorbid depression and 61.7% for insomnia comorbid anxiety) and diabetes (34.0% for insomnia comorbid depression and 32.1% for insomnia comorbid anxiety; see Table 1). The prevalence of patients with these conditions across different age groups is shown in Supplementary Fig. 2.

**Table 1:** Characteristics of patients with insomnia combined with depressive or anxiety disorders at primary care facilities, 2022

Variables	Insomnia comorbid depression	Insomnia comorbid anxiety
Number of patients (*n*)	842	1014
Mean age, years (s.d.)	68.8 (12.3)	66.5 (12.3)
Age, years, *n* (%)		
** **18–44	33 (3.9)	52 (5.1)
** **45–64	267 (31.7)	371 (36.6)
** **65–74	289 (34.3)	367 (36.2)
** **75–84	149 (17.7)	135 (13.3)
** **≥85	105(12.5)	89 (8.8)
Gender, *n* (%)		
** **Female	523 (62.1)	641 (63.2)
** **Male	319 (37.9)	373 (36.8)
Insurance type, *n* (%)		
** **Medical insurance	802 (95.2)	966 (95.3)
** **Free medical care	24 (2.9)	35 (3.5)
** **Out of pocket	28 (3.3)	20 (2.0)
** **Other	2 (0.2)	1 (0.1)
Common complications, *n* (%)		
** **Hypertension	561 (66.6)	626 (61.7)
** **Diabetes	286 (34.0)	325 (32.1)
** **Arthritis	45 (5.3)	37 (3.6)
** **COPD	30 (3.6)	31 (3.1)
** **Dementia	12 (1.4)	3 (0.3)
** **Parkinson’s disease	14 (1.7)	16 (1.6)

COPD, chronic obstructive pulmonary disease.

### Distribution of prescribed psychotropic medications by drug classes

**Fig. 1:**
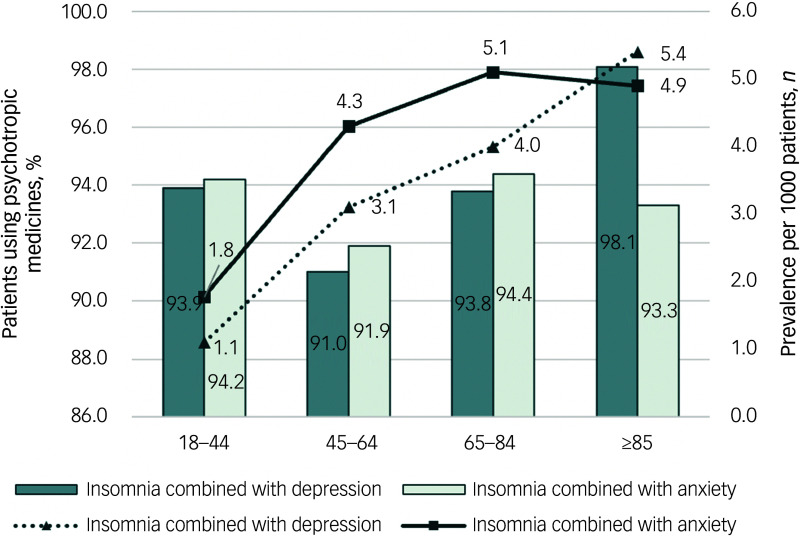
Prevalence of insomnia comorbid with depressive or anxiety disorders and utilisation of psychotropic medications among patients in primary healthcare facilities in Beijing, 2022.

**Fig. 2:**
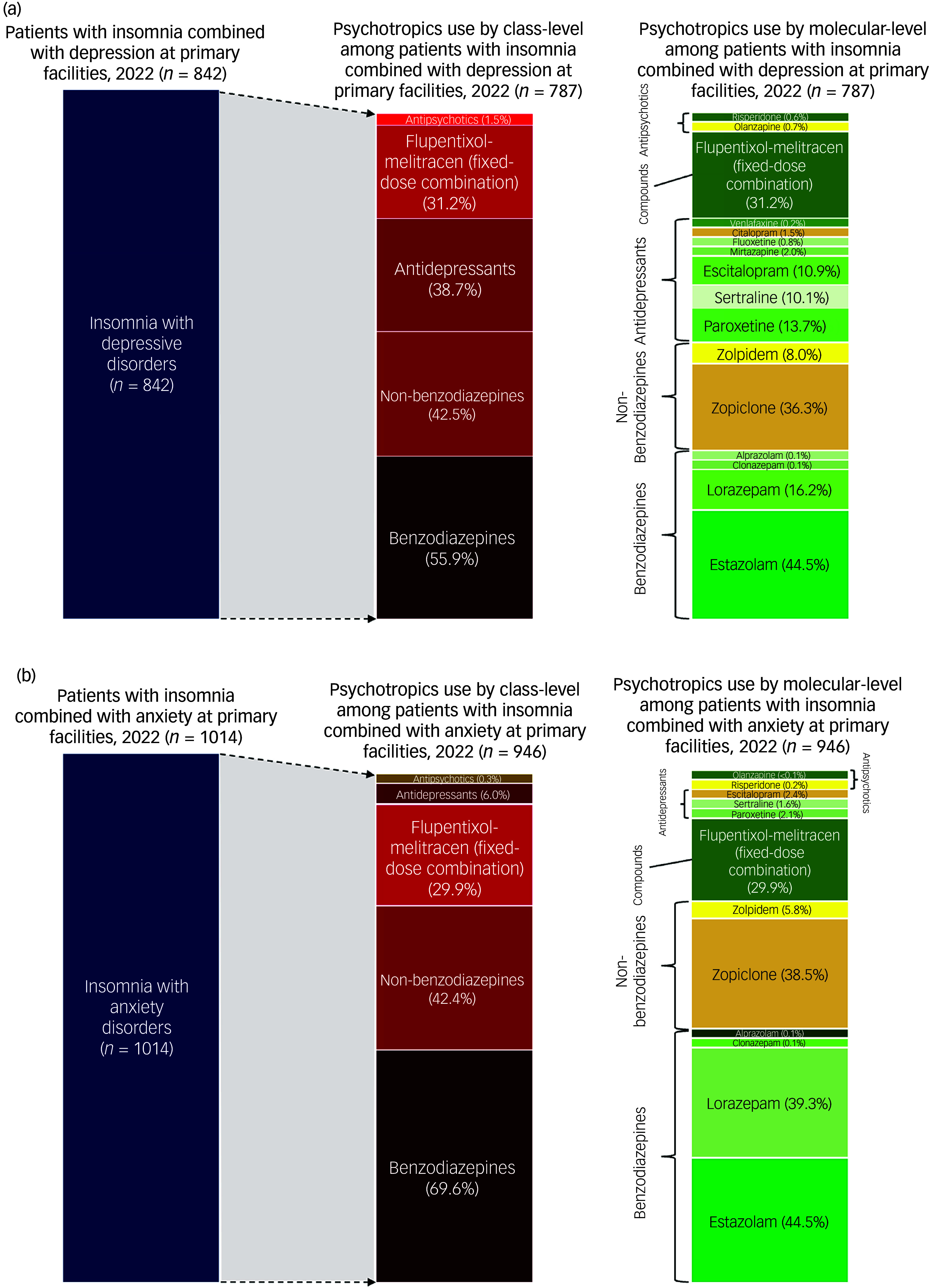
Distribution of psychotropic medications among patients with (a) insomnia and depressive disorders or (b) insomnia and anxiety disorders, in primary healthcare facilities in Beijing, 2022.

Across both comorbid groups, more than 90% of patient visits included at least one psychotropic medication, with minimal variation across age groups (Fig. 1). Benzodiazepines were the most frequently prescribed classes (55.9% for insomnia comorbid depression and 69.6% for insomnia comorbid anxiety), followed by non-benzodiazepines (42.5% for insomnia comorbid depression and 42.4% for insomnia comorbid anxiety). SSRIs and flupentixol-melitracen were also commonly used (SSRIs: 36.6% for insomnia comorbid depression and 6.0% for insomnia comorbid anxiety; flupentixol-melitracen: 31.2% for insomnia comorbid depression and 29.9% for insomnia comorbid anxiety). Antidepressants with sedative effects were prescribed infrequently (<5%) (Fig. 2(a) and (b)). The most commonly prescribed benzodiazepines were estazolam (44.5% for insomnia comorbid depression and 44.5% for insomnia comorbid anxiety) and lorazepam (16.2% for insomnia comorbid depression and 39.3% for insomnia comorbid anxiety), whereas zopiclone (36.3% for insomnia comorbid depression and 38.5% for insomnia comorbid anxiety) and zolpidem (8.0% for insomnia comorbid depression and 5.8% for insomnia comorbid anxiety) were most frequently used non-benzodiazepines. SSRIs such as paroxetine, sertraline and escitalopram were more often prescribed for insomnia patients with depression (paroxetine: 13.7%; sertraline: 10.1%; escitalopram: 10.9%) than for those with anxiety (paroxetine: 2.1%; sertraline: 1.6%; escitalopram: 2.4%).

### Guideline-recommended pharmacotherapy

**Fig. 3:**
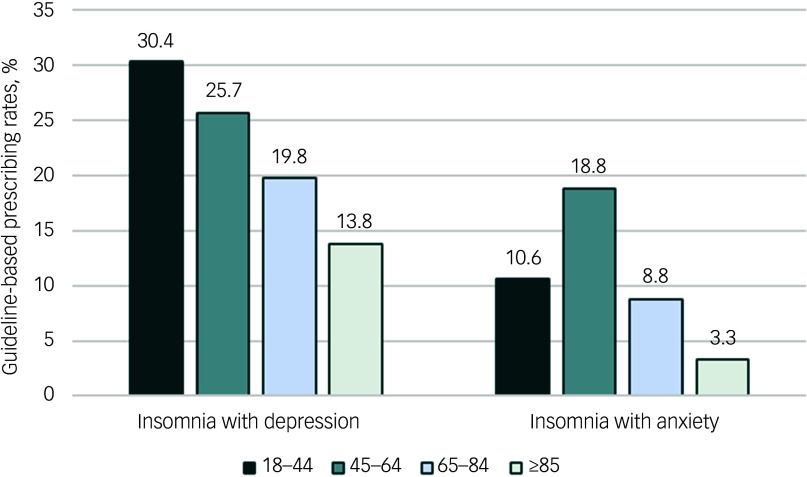
Proportion of guideline-recommended prescribing for insomnia combined with depressive or anxiety disorders by patient age group in primary care facilities in Beijing, 2022.

Approximately 24.8 and 17.0% of patients diagnosed with insomnia and either depressive or anxiety disorders received combined therapy, respectively. For patients with insomnia and depression, 29.9% received guideline-recommended pharmacotherapy, whereas this was only 11.5% for those with insomnia and anxiety (Table 2). We also observed a decreasing trend in the prescribing rate of guideline-recommended pharmacotherapy among older adults with insomnia comorbid depression (Fig. 3).

**Table 2:** Guideline recommendations and prescribing patterns of the pharmacotherapy among patients with insomnia combined with depressive or anxiety disorders in primary care facilities in Beijing, 2022

Treatment therapy	Insomnia combined with depression		Insomnia combined with anxiety	
	Prescriptions of each pattern, *n* (%)^a^	Recommendations of guidelines^7^	Prescriptions of each pattern, *n* (%)^a^	Recommendations of guidelines^7^
All visits containing one or more psychotropic medications	5404 (100.0)		6997 (100.0)	
Monotherapy	4065 (75.2)		5805 (83.0)	
** **SSRIs/SNRIs	673 (12.5)	√	63 (0.9)	√
** **Antidepressants with sedative effects^b^	40 (0.7)	√	0 (0.0)	√
** **Benzodiazepines	1544 (28.6)	×	3159 (45.1)	⍻
** **Non-benzodiazepines	1387 (25.7)	×	1843 (26.3)	×
** **Fixed-dose combination^c^	414 (7.7)	√	739 (10.6)	√
** **Antipsychotics	7 (0.1)	×	1 (<0.1)	×
Combined therapy (two medications)	497 (9.2)		551 (7.9)	
** **SSRIs/SNRIs + antidepressants with sedative effects	1 (<0.1)	√	0 (0.0)	√
** **SSRIs/SNRIs + benzodiazepines	203 (3.8)	×	27 (0.4)	⍻
** **SSRIs/SNRIs + non-benzodiazepines	0 (0.0)	⍻	0 (0.0)	×
** **SSRIs/SNRIs + fixed-dose combination	2 (<0.1)	×	0 (0.0)	×
** **SSRIs/SNRIs + antipsychotics	14 (0.3)	×	0 (0.0)	×
** **Antidepressants with sedative effects + benzodiazepines	21 (0.4)	⍻	0 (0.0)	⍻
** **Antidepressants with sedative effects + non-benzodiazepines	8 (0.1)	⍻	0 (0.0)	×
** **Antidepressants with sedative effects + antipsychotics	0 (0.0)	×	0 (0.0)	×
** **Benzodiazepines + non-benzodiazepines	40 (0.7)	×	258 (3.7)	×
** **Bbenzodiazepines + fixed-dose combination	128 (2.4)	×	103 (1.5)	×
** **Benzodiazepines + antipsychotics	2 (<0.1)	×	2 (<0.1)	×
** **Non-benzodiazepines + fixed-dose combination	48 (0.9)	⍻	92 (1.3)	×
** **Non-benzodiazepines + antipsychotics	1 (<0.1)	×	0 (0.0)	×
** **Benzodiazepines + benzodiazepines	29 (0.5)	×	67 (1)	×
** **Non-benzodiazepines + non-benzodiazepines	0 (0.0)	×	2 (<0.1)	×

SSRIs, selective serotonin reuptake inhibitors; SNRIs, selective norepinephrine reuptake inhibitors; √, recommended; ×, not recommended or not mentioned; ⍻, conditional recommended.

a. Denominator: the number of visits with at least one psychotropic medication of sample patients.

b. Mirtazapine, trazodone.

c. Flupentixol-melitracen.

## Discussion

Our findings revealed that the prescribing rate of guideline-recommended pharmacotherapy was low for patients with insomnia comorbid depressive or anxiety disorders at primary healthcare facilities in China. Benzodiazepines and non-benzodiazepines were the most commonly prescribed medications, despite clinical guidelines prioritising antidepressants with sedative effects, SSRIs and SNRIs, which were less frequently used.^7^

The prescribing rates of psychotropic medications for patients with insomnia comorbid with depression or anxiety exceeded 90% overall and across different age groups. Psychotherapy, such as CBT, has been demonstrated to be effective for common mental disorders (e.g. insomnia, depression, anxiety, solely or comorbid), and is recommended as first-line treatment in current clinical guidelines.^9,10,25,26,^ However, access to psychotherapy remains extremely limited worldwide particularly in primary healthcare facilities, because of inadequate resources and a shortage of trained therapists.^11,12^ Given these constraints, patients with mental disorders were frequently treated only pharmacologically.^12,14^ Enhancing access to psychotherapy may help reduce unnecessary psychotropic medication use and mitigate associated adverse effects and drug-drug interactions, which is particularly pertinent to older persons.^22^

Our findings highlighted that only 29.9% of patients with comorbid insomnia and depression and 11.5% of those with comorbid anxiety received treatments aligned with guideline recommendations. We observed high overall use of psychotropic medications across all age groups, but guideline-recommended prescribing rates were lower among adults aged ≥65 years, particularly among those with insomnia and depressive disorder. In older populations, sedative hypnotics should generally be avoided because they are associated with falls, delirium, cognitive impairment and dependence.^27^ Given the high prevalence of multimorbidity and polypharmacy in older adults, primary care should routinely review medications and screen for cumulative sedative and anticholinergic burden. Expanding access to CBT for insomnia and, when syndromic depressive or anxiety disorders are present, considering antidepressants with sleep-promoting properties may reduce reliance on chronic hypnotics in this high-risk group.

Another major concern is the high prescribing rates of non-benzodiazepines and benzodiazepines, both of which should be used with caution according to the Chinese guideline.^7^ However, our results showed that non-benzodiazepines were prescribed for 42.5% of patients with insomnia comorbid depression and 42.4% of those with comorbid anxiety, representing substantial inappropriate use. Moreover, although benzodiazepines were considered a last resort by guidelines because of their high risk of inducing cognitive dysfunction and falls,^28–30^ they were prescribed at alarmingly high rates in both conditions (55.9 and 69.6%), far exceeding rates reported in the UK (14.5–27.1%).^22^ This pattern of high non-benzodiazepine and benzodiazepine use, particularly noted in patients aged >85 years, has also been reported in our previous research focusing on insomnia treatment in primary care.^14^ This pervasive issue may stem from clinicians’ limited knowledge of guideline recommendations in managing comorbid common mental disorders.^14^ Tailored training programmes are therefore necessary to promote evidence-based treatment at primary healthcare facilities and reduce excessive use of benzodiazepines, especially among older adults.

The prevalent use of benzodiazepines or non-benzodiazepines as monotherapy in treating comorbid conditions suggests an oversight of depressive and anxiety symptoms by primary clinicians. This issue may primarily arise from primary care physicians’ unfamiliarity with the guidelines as discussed earlier, but it might also be influenced by patients’ expectations.^31^ Some patients who initiated appropriate treatment at specialty hospitals or tertiary hospitals may seek stronger sedative hypnotics at primary care. This highlights the need for primary clinicians to be trained to meticulously review patients’ psychotropic medication use during consultations and make necessary medication reconciliation to ensure more appropriate prescribing and patient safety.^32^ Establishing a shared network system among different healthcare levels to track diagnosis and treatment history could facilitate more comprehensive care.^33^ Future studies using regional databases that link different levels of healthcare institutions are need to better understand cross-setting differences in pharmacotherapy for insomnia comorbid with depression or anxiety.

Our research also showed prevalent use of flupentixol-melitracen in primary healthcare facilities in China, a pattern not observed in other countries.^22,34^ Flupentixol-melitracen is a fixed-dose combination of a typical antipsychotic and melitracen, a tricyclic antidepressant. It has not been marketed in many high-income countries (e.g. USA, Canada, Japan, Denmark), and was banned by India’s health ministry in 2015 because of safety concerns regarding fixed-dose combinations of psychotropic agents.^35^ Despite these international reservations, flupentixol-melitracen continues to be widely used in China, where it has been marketed since the late 1990s. Although Chinese guidelines recognise its efficacy for treating depression and anxiety, they offer limited guidance on its use in managing insomnia comorbid with depression or anxiety. The substantial use of this combination in the absence of definitive and conclusive evidence raises concerns about potential harms. Rigorous clinical research is urgently needed to establish clear evidence to inform and refine future treatment recommendations. Further, Chinese guidelines should provide more specific and concrete recommendations for flupentixol-melitracen to support safer clinical practice and improve patient outcomes.

Several limitations of this study should be noted. First, the data sources contained only primary healthcare facilities, whereas the treatment of depression and anxiety might predominantly occur in specialised or tertiary hospitals in China. This may explain the relatively small sample size of patients with insomnia comorbid depression or anxiety in our study, limiting its generalisability. Future region-wide, cross-setting linked studies are needed to assess duration and trajectories of care. Second, potential inaccuracy or inconsistency of diagnostic coding in the database may have affected case identification and inclusion. However, we also used the corresponding Chinese text diagnoses recorded in the electronic health records, which helped to partially mitigate this uncertainty in coding. Third, the study was conducted in Dongcheng, a district in central Beijing with higher economic status and superior medical resources. Consequently, the findings may not be fully representative of the broader population of patients in primary care across Beijing. Fourth, we restricted our study period to 2022 to maximise data completeness and coding stability. A single-year analysis limits assessment of trends such as seasonality or secular shifts.

In conclusion, the prescribing rate of guideline-recommended pharmacotherapy for patients with insomnia comorbid with depressive or anxiety disorders was considerably low in Chinese primary healthcare facilities, primarily because of the excessive use of benzodiazepines and non-benzodiazepines. These findings highlight the need to reduce the use of high-risk medications and to facilitate evidence-based treatment for complex mental health conditions at primary care, especially for older adults.

## Supporting information

Fu et al. supplementary material Fu et al. supplementary material

## Data Availability

The data that support the findings of this study are available from the corresponding author, X.G., upon reasonable request.

## Supplementary material

The supplementary material is available online at https://doi.org/10.1192/bjo.2025.10967
