# Supplementary material for: Psychotropic medication prescribing for patients with insomnia comorbid with depressive or anxiety disorders in primary healthcare facilities in Beijing
Source: BJPsych Open. 2026 Feb 5;12(2):e55. doi: 10.1192/bjo.2025.10967 (PMC12926887; doi:10.1192/bjo.2025.10967)
Supplement: Fu et al. supplementary material [file S2056472425109678sup001.docx]

## Psychotropic Medication Prescribing for Patients with Insomnia Comorbid with Depression or Anxiety Disorder in Primary Healthcare Facilities in Beijing

## Supplement

**eFigure 1.** Study Flow Diagram

**eTable 1.** List and Classification of Psychotropic Medications according to the Anatomical Therapeutic Chemical (ATC) classifications and Related Clinical Guidelines

**eTable 2.** Key Recommendations from Chinese Clinical Guidance


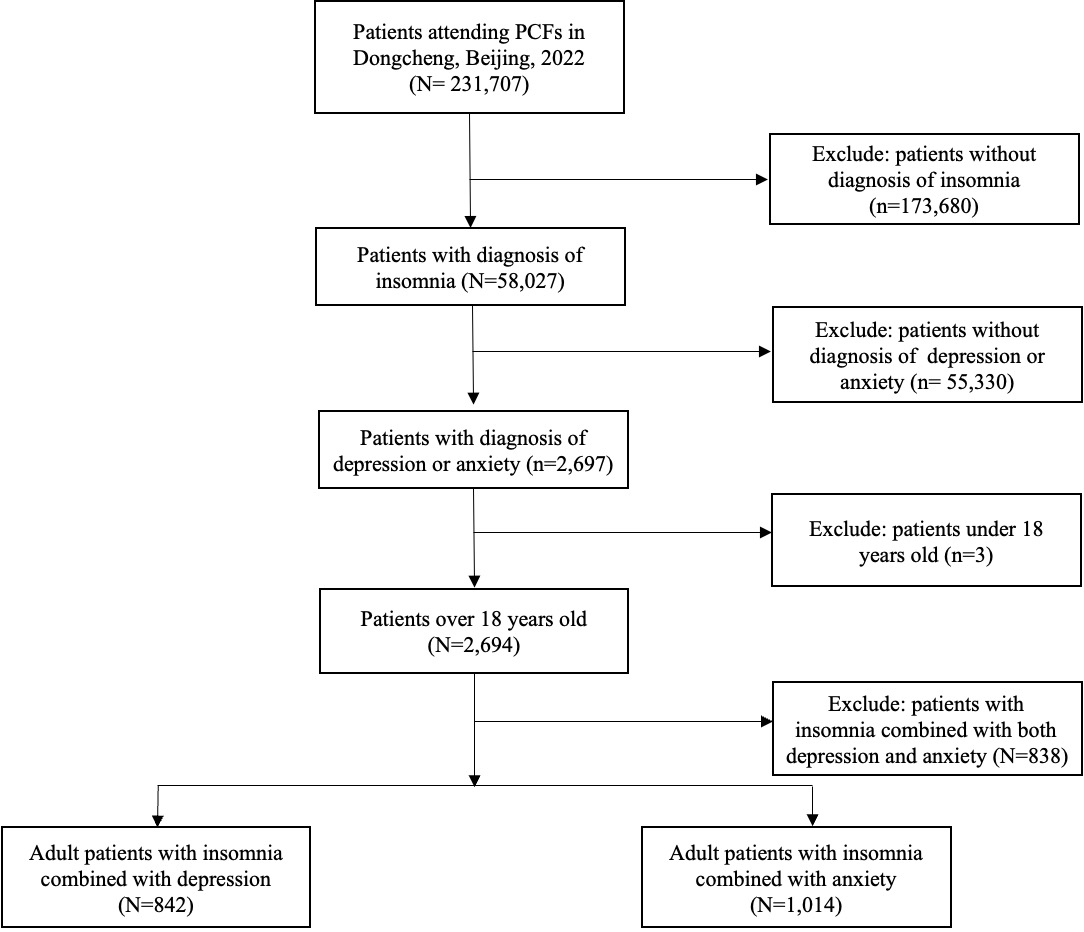


### eFigure 1. Study Flow Diagram

### eTable 1. List and Classification of Psychotropic Medications according to the Anatomical Therapeutic Chemical (ATC) classifications and Related Clinical Guidelines

| **Classification** | **Medications** |
| --- | --- |
| **Hypnotics and Sedatives and Other Anxiolytics** | ***Benzodiazepines:*** Alprazolam, Clonazepam, Diazepam, Estazolam, Flurazepam, Lorazepam, Midazolam, Nitrazepam, Oxazepam, Quazepam, Temazepam, Triazolam  ***Nonbenzodiazepine, benzodiazepine receptor agonist hypnotics:*** Eszopiclone, Zopiclone, Zaleplon, Zolpidem  ***Other anxiolytics:*** Hydroxyzine, Buspirone, Tandospirone |
| **Antidepressants** | ***Selective serotonin reuptake inhibitors:*** Citalopram, Escitalopram, Fluoxetine, Fluvoxamine, Paroxetine, Sertraline  ***Serotonin and Noradrenalin Reuptake Inhibitors:*** Duloxetine, M**ilnacipran**, Venlafaxine  ***Tricyclicantidepressants:*** Amitriptyline, Doxepin, Imipramine  ***Monoamine oxidase A inhibitor:*** Moclobemide  ***Other antidepressants:*** Agomelatine, Bupropion, **Mirtazapine***, Maprotiline, Mianserin, Riboxetine, Tianeptine, **Trazodone***, Vortioxetine, Flupentixol-melitracen |
| **Antipsychotics** | Aripiprazole, Asenapine, Chlorpromazine, Clozapine, Droperidol, Fluphenazine, Haloperidol, Iloperidone, Loxapine, Lurasidone, Olanzapine, Paliperidone, Perphenazine, Pimozide, quetiapine, Risperidone, Sulpiride, Thioridazine, Thiothixene, Trifluoperazine, Ziprasidone |
| **Fixed-dose combination** | Flupentixol/melitracen |

* Antidepressant with sedative effects.

### eTable 2. Key Recommendations from Chinese Clinical Guidance

| **Condition** | **Guideline recommendation** | **Pharmacotherapy** | **Our mapping in analyses** |
| --- | --- | --- | --- |
| Insomnia comorbid depressive disorder | CBT-I is first-line for chronic insomnia; initiate antidepressants for moderate to severe depression; when insomnia is prominent, consider agents with sedative properties; BZDs/non-BZDs used short-term only; avoid long-term BZDs/non-BZDs use. | SSRIs/SNRIs as first-line; NaSSA (i.e., mirtazapine)/SARI (trazodone) as alternatives/adjuncts; BZDs/non-BZDs as short-term adjuncts. | **Recommended:** SSRI/SNRI/NaSSA/SARI. **Conditional recommended:** BZDs/non-BZDs adjunct. |
| Insomnia comorbid anxiety disorder | Disorder-specific CBT first-line for mild; pharmacotherapy (SSRIs/SNRIs) for moderate to severe; BZDs/non-BZDs may be used short-term as adjuncts for acute anxiety/insomnia; avoid long-term BZDs/non-BZDs use. | SSRIs/SNRIs as first-line for moderate to severe; BZDs/non-BZDs as short-term adjuncts. | **Recommended:** SSRI/SNRI NaSSA/SARI. **Conditional recommended:** short-term BZDs/non-BZDs adjunct. |
| Insomnia + depression + anxiety (tri-morbid) | Guidance emphasizes individualized treatment addressing both psychiatric disorders and insomnia; CBT-I + antidepressant ± anxiolytic/hypnotic may be tailored to symptom profile and risk. | No single uniform algorithm; short-term BZDs/non-BZDs adjuncts may be used cautiously; prioritize antidepressants for depression or anxiety. | No analyses. |

1. “Short-term” refers to brief, adjunctive use during the acute phase (e.g., typically ≤4 weeks) with tapering plans; prolonged or continuous use beyond this window is treated as long-term in our classification.
2. CBT-I/CBT recommendations are summarized here for context; psychotherapy was not observed in the EHR and not used for classification.

**Abbreviations.** CBT-I, cognitive behavioral therapy for insomnia; CBT, cognitive behavioral therapy; SSRI, selective serotonin reuptake inhibitor; SNRI, serotonin-noradrenaline reuptake inhibitor; NaSSA, noradrenergic and specific serotonergic antidepressant; SARI, serotonin antagonist and reuptake inhibitor; BZD, benzodiazepine.
